# ENERGI-F703 gel, as a new topical treatment for diabetic foot and leg ulcers: A multicenter, randomized, double-blind, phase II trial

**DOI:** 10.1016/j.eclinm.2022.101497

**Published:** 2022-07-10

**Authors:** Jui-Yung Yang, Cha-Chun Chen, Shun-Cheng Chang, Jiun-Ting Yeh, Hui-Fu Huang, Hwang-Chi Lin, Shang-Hsi Lin, Yu-Hsien Lin, Lin-Gwei Wei, Tom J. Liu, Shih-Yuan Hung, Hui-Mei Yang, Hui-Hsiu Chang, Chih-Hsin Wang, Yuan-Sheng Tzeng, Chieh-Huei Huang, Chang-Yi Chou, Ying-Sheng Lin, Shih-Yi Yang, Han-Min Chen, Jiun-Tsai Lin, Yi-Fang Cheng, Guang-Huar Young, Chun-Fang Huang, Ya-Chun Kuo, Niann-Tzyy Dai

**Affiliations:** a Division of General Plastic Surgery, Taipei Chang Gung Memorial Hospital, Taipei, Taiwan; b Division of Plastic Surgery, Shin Kong Memorial Wu Ho-Su Hospital, Taipei, Taiwan; c Division of Plastic Surgery, Shuang Ho Hospital, New Taipei, Taiwan; d Division of Trauma Plastic Surgery, Linkou Chang Gung Memorial Hospital, Taoyuan, Taiwan; e Division of Plastic Surgery, National Taiwan University Hospital, Taipei, Taiwan; f Division of Plastic Surgery, Taoyuan Armed Forces General Hospital, Taoyuan, Taiwan; g Division of Plastic Surgery, National Taiwan University Hospital Yunlin Branch, Yunlin, Taiwan; h Division of Endocrinology and Metabolism, Linkou Chang Gung Memorial Hospital, Taoyuan, Taiwan; i Division of Plastic and Reconstructive Surgery, Tri-Service General Hospital, Taipei, Taiwan; j Energenesis Biomedical Co. Ltd, Taipei, Taiwan

**Keywords:** Diabetic foot ulcer, Diabetic wound healing, Adenine, Adenosine triphosphate, ATP

## Abstract

**Background:**

Diabetic foot and leg ulcers are a major cause of disability among patients with diabetes mellitus. A topical gel called ENERGI-F703, applied twice daily and with adenine as its active pharmaceutical ingredient, accelerated wound healing in diabetic mice. The current study evaluated the safety and efficacy of ENERGI-F703 for patients with diabetic foot and leg ulcers.

**Methods:**

This randomized, double-blind, multicenter, phase II trial recruited patients from eight medical centers in Taiwan. Patients with intractable diabetic foot and leg ulcers (Wagner Grade 1–3 without active osteomyelitis) were randomly assigned (2:1) to receive topical ENERGI-F703 gel or vehicle gel twice daily for 12 weeks or until complete ulcer closure. The investigator, enrolled patients and site personnel were masked to treatment allocation. Intention to treat (ITT) population and safety population were patient to primary analyses and safety analyses, respectively. Primary outcome was complete ulcer closure rate at the end of treatment. This trial is registered with ClinicalTrials.gov, number NCT02672436.

**Findings:**

Starting from March 15^th^, 2017 to December 26^th^, 2019, 141 patients were enrolled as safety population and randomized into ENERGI-F703 gel (n = 95) group or vehicle gel (n = 46) group. In ITT population, ENERGI-F703 (n = 90) and vehicle group showed ulcer closure rates of 36.7% (95% CI = 26.75% - 47.49%) and 26.2% (95% CI = 13.86% - 42.04%) with difference of 9.74 % (95 % CI = -6.74% - 26.23%) and 25% quartiles of the time to complete ulcer closure of 69 days and 84 days, respectively. There were 25 (26.3%) patients in ENERGI-F703 group and 11 (23.9%) patients in vehicle group experiencing serious adverse events and five deaths occurred during the study period, none of them related to the treatment.

**Interpretation:**

Our study suggests that ENERGI-F703 gel is a safe and well-tolerated treatment for chronic diabetic foot and leg ulcers. Further studies are needed to corroborate our findings in light of limitations.

**Funding:**

Energenesis Biomedical Co., Ltd.

**Research in context**

**Evidence before this study**

We searched PubMed on March 24, 2021, using the terms "adenine, ulcer" or "adenine, wound healing", without exclusions of language, date, or article type. In total, 142 and 129 papers were identified, respectively. No studies were relevant to the effect of adenine on wound healing or ulcer management except for two preclinical studies reported by our team. In this study, the efficacy and safety of adenine were further evaluated in a double-blinded and randomized clinical trial focused on patients with diabetic foot ulcers.

**Added value of this study**

Our study suggests that a gel containing adenine is safe to use and could help the healing process of diabetic skin wounds. To the best of our knowledge, this is the first clinical study to assess the effect of adenine on wound healing in patients with diabetic foot ulcers.

**Implications of all the available evidence**

The efficacy of ENERGI-F703 gel will be further confirmed in future study with a larger population.

Alt-text: Unlabelled box

## Introduction

Diabetes mellitus (DM) is among the most common metabolic disorders globally, with sustained high prevalence in most Western countries and rapidly growing prevalence in Asia and other regions. The estimated total number of patients reached 463 million in 2019 and is projected to exceed 700 million by 2045.^1^ While disruption of blood glucose control due to deficient insulin signaling is the defining clinical characteristic of DM, it is the longer-term cardiovascular, neural, and renal complications that are the main causes of disability and mortality. Foot and leg ulcers are one of the many problems caused by poorly controlled diabetes. Ulcers that do not heal can result in amputations of toes, parts of the foot, or the lower leg.^2^, ^3^, ^4^, ^5^ Every year, particularly, about 1%–4% of patients with diabetes develop diabetic foot ulcers (DFU)^2^^,^^6^; moreover, about 25% of DM patients will experience at least one foot ulcer during the disease course.^6^^,^^7^ The overall rate of hospital discharge for new lower extremity amputations (LEAs) was about 4.3 per 1,000 people with diabetes compared with a rate of about 0.3 per 1,000 in the general population in 2005, and the 5-year mortality rate of LEA recipients exceeds 70%.^7^ In the U.S., around 130,000 LEAs are conducted annually on patients with diabetes,^8^ which collectively put an enormous strain on primary healthcare and social resources. Thus, effective treatments to promote diabetic foot and leg ulcers healing may reduce LEA risk and early mortality.

Current treatments for diabetic foot and leg ulcers include the standards of care for wound healing, such as surgical debridement, maintenance of a moist wound environment with dressing, wound off-loading, vascular assessment, and treatment of active infection, in combination with strict DM management, especially glycemic control. Additionally, there are several adjuvant therapies in use or under study to improve diabetic foot and leg ulcers outcome, such as hyperbaric oxygen therapy, negative-pressure wound therapy, acellular dermal matrix treatment, skin grafting, and local administration of various growth factors.^9^ Among growth factor treatments, recombinant platelet-derived growth factor sold under the brand name Regranex® is the only FDA-approved drug for DFU. However, this drug has not met expectations for cost-effectiveness and efficacy.^10^

To develop a more efficient and safe way to treat diabetic foot and leg ulcers, we turned our focus on methods that can stimulate cellular activity to invigorate wound healing process. Adenosine triphosphate (ATP) is known to promote the haemostatic, immune, and local cellular responses required for tissue repair and regeneration. For instance, addition of an ATP generating system can induce the proliferation and M2 polarization of macrophages, which enhance the release of cytokines and chemokines and stimulate stem cell proliferation.^11^ Indeed, intracellular ATP delivery systems have proven effective for enhancing the healing of diabetic skin wounds in animal models.^12^, ^13^, ^14^

We have demonstrated previously that exogenous application of adenine can statistically significantly increase intracellular ATP concentration by expanding the intracellular adenylate pool and the induction of 5′-adenosine monophosphate (5′ AMP)-activated protein kinase (AMPK) phosphorylation.^15^^,^^16^ Based on this result, we formulated the adenine-containing ENERGI-F703 Gel and subsequently found that it shortened the time to complete wound closure in a diabetic mouse model.^16^ With well-established safety profile, the addition of adenine to citrate-phosphate-dextrose (CPD)-blood solution is used in many countries to improve the preservation of erythrocytes during storage.^17^ Also, adenine has been approved by Japanese authorities for use in oral and injected preparations to treat leucopoenia in Japan.^18^ Therefore, an adenine-based topical gel may be a safe and effective treatment for facilitating wound healing in diabetic foot and leg ulcers patients. In this report, we aimed to evaluate the efficacy and safety of ENERGI-F703 Gel in diabetic foot and leg ulcers patients with a randomized, double-blind, vehicle-controlled, parallel and multicenter phase II trial.

## Methods

### Study design

This was a randomized, double-blind, vehicle-controlled, parallel, multicenter study involving eight centers in Taiwan, including Tri-Service General Hospital (ethics committee reference number: 2-105-01-008), National Taiwan University Hospital (ethics committee reference number: 201612129MSD), Chang Gung Medical Foundation-Taipei (ethics committee reference number: 201700333A4), Shin Kong Wu Ho-Su Memorial Hospital (ethics committee reference number: 20161204C), Taoyuan Armed Forces General Hospital (ethics committee reference number: 2-105-01-008), Chang Gung Medical Foundation-Linkou (ethics committee reference number: 201700333A4), National Taiwan University Hospital-Yunlin (ethics committee reference number: 201612129MSD), Shuang Ho Hospital (ethics committee reference number: N201902051). The study was conducted in accordance with the principle of good clinical practice and the Declaration of Helsinki, and approved by the United States Food and Drug Administration (FDA), the Taiwan Food and Drug Administration, and the Institutional Review Boards of each participating center. The trial is also registered with ClinicalTrial.gov (Identifier: NCT02672436).

### Study population

The study population consisted of DM patients 20 years of age or older diagnosed with diabetic foot and leg ulcers, one of which (see selection criteria below) was selected by an investigator as the treatment target. All patients provided written informed consent prior to enrollment. The inclusion criteria were age of at least 20 years, DM defined as currently under DM treatment or naïve with duplicated HbA1c over 6.5% and fasting plasma glucose over 126 mg/dL measured at least one week apart before screening, and at least one cutaneous ulcer on the foot, including ulcers on the lower legs, that had not healed for at least 4 weeks. The largest such ulcer was selected as the treatment target. If the patient exhibited two or more large ulcers, the most severe according to the Wagner Grading System was selected. If there were two or more ulcers with the same size and Grade, the one with longest duration was selected. All target ulcers were classified as Grade 1–3 and ranged in size from 1 cm^2^–36 cm^2^. Patients with Grade 3 ulcer were enrolled only if the target ulcer was under control after debridement (as judged by the investigator) and showing no signs of local osteomyelitis and soft tissue infection. Patients were excluded if presented with a target ulcer with infection, active osteomyelitis, a target ulcer decrease by at least 30% in size after 2 weeks of standard-of-care-only treatment and any other recorded regular therapy either before the screening visit or after completing the Initial Phase. Other exclusion criteria were poor nutritional status (albumin < 2 g/dL), poor diabetic control (HbA1c > 12%), leukocyte count < 2,000/mm^3^, and abnormal liver function as indicated by serum aspartate aminotransferase (AST) or alanine aminotransferase (ALT) > 3 times the upper limit of the normal range within 14 days before the screening visit or 28 days before randomization. Patients requiring systemic corticosteroids, immunosuppressive or chemotherapeutic agents, those with known or suspected hypersensitivity to any ingredients in the study product or vehicle were also excluded, as were patients with coronary heart disease or history of myocardial infarction, history of coronary artery bypass graft, or percutaneous transluminal coronary angioplasty within 3 months prior to the study. Pregnant, lactating, and premenopausal women with childbearing potential not taking reliable contraceptives during the study period were also excluded. Finally, candidates were excluded for the following conditions: a. ankle brachial index (ABI) < 0.4 or b, ABI between 0.4 and 0.6 (inclusive) and not receiving appropriate treatment for venous and arterial insufficiency, enrolment in any investigational drug trial within 4 weeks before entering this study, and any condition that may enhance treatment risk as judged by the investigator.

### Randomization and blinding

Eligible patients were enrolled and assigned randomization numbers in sequence by trial investigator for allocation into ENERGI-F703 Gel (0.02% adenine) and vehicle gel groups in a 2:1 ratio. Randomization codes were generated using a permuted block randomization method stratified by baseline target ulcer size (<16 cm^2^ or ≥16 cm^2^) and trial center. Randomization was performed by the trial biostatistician who did not partake in the clinical operations of the trial. All participants, investigators and personnel of study site and sponsor were blinded to treatment allocation. ENERGI-F703 Gel and vehicle gel were identical in all aspects (e.g., color, odor, appearance of the vial content or package) except for the active ingredient (0.02% adenine) to achieve double blinding.

### Procedures

The trial contained a screen period of 3 weeks, a treatment period of 12 weeks and an observation period of 12 weeks (or 4 weeks for patients with incomplete ulcer closure). Amid treatment period, eligible patients received either ENERGI-F703 Gel or vehicle gel on target ulcers twice daily for 12 weeks or until confirmed complete ulcer closure, whichever came first. Once target ulcers developed infections and need local or intravenous antibiotics treatment, the patients were withdrawn from the study. After the treatment period, patients were monitored for target ulcer recurrence and safety.

In addition to the investigational drug or vehicle, patients with incomplete closure received current standard-of-care procedures throughout the study, including ulcer debridement, wound cleansing and maintenance of a moist wound environment. Investigators or study-trained nurses also educated patients on standard-of-care procedures to enable them to perform target ulcer care at home.

All patients were allowed routinely used medications or treatments excluding prohibited from taking systemic corticosteroids, immunosuppressive, or chemotherapeutic agents, hyperbaric oxygen therapy, or any treatment that may affect investigational drug evaluation as judged by the investigator.

### Outcomes

The primary efficacy endpoint was the proportion of patients with complete ulcer closure at the end of the treatment period. Complete ulcer closure was defined as 100% skin re-epithelialization without drainage or dressing requirements as confirmed on two consecutive study visits, which was two weeks apart.

The secondary efficacy endpoint was the time to complete ulcer closure within the treatment period. Target ulcer evaluation was performed at each visit throughout the study even among patients completely healed. At each evaluation, target ulcers were photographed together with an ulcer measuring ruler, and the surface area estimated using ImageJ (National Institutes of Health, http://rsb.info.nih.gov/ij/download.html, (ImageJ version: v1.5i_32bit or v1.5i_64bit)

For safety evaluation, information was collected on adverse events, adverse drug reactions, serious adverse events, and serious adverse drug reactions at each visit. Safety was also monitored by laboratory tests, physical examinations, vital signs, and 12-lead electrocardiogram (ECG) at indicated visits. AEs were listed by Medical Dictionary for Regulatory Activities (version 20.0) system organ class and preferred terms. Severity of AEs and relationships between AEs and the investigation product were appraised by investigators.

### Statistical analysis

Three populations were defined for statistical analyses, a safety population, intention-to-treat (ITT) population and per protocol (PP) population. The safety population included all randomized patients who received at least one dose of the investigational drug. Among the safety population, patients who fulfilled all enrolment criteria were defined as the ITT population, while the PP population was defined as the ITT population subset with at least 75% treatment adherence and not receiving any prohibited medication or therapy during the treatment period. Efficacy endpoints were analyzed on the ITT and PP populations, demographics and baseline characteristics for the ITT population, and safety endpoints for the safety population. The sample size in the study was determined by preclinical study of 25% drug efficacy rate and statistical power of 0.7 to achieve adequate evaluation of the efficacy and safety. There was an interim analysis assessing the primary endpoint to evaluate the continuance of study recruitment and increase of patient number.

Descriptive statistics was provided for demographic and baseline clinical characteristics and all endpoint analyses. Wilcoxon test was performed at the request of a reviewer. Frequency table was provided for categorical data, while mean, standard deviation, median, IQR and 95% two sided confidence interval were presented for treatment difference. Ulcer closure rates were compared between treatment groups using the Cochran-Mantel-Haenszel (CMH) test. Ulcer closure times were estimated by the Kaplan-Meier method and compared between groups by log-rank test. To identify the population demonstrating greatest ENERGI-F703 Gel efficacy, the efficacy endpoints were post-hoc analyzed by sub-grouping patients according to baseline ulcer Grade and size. DFUs were defined by the International Working Group on the Diabetic Foot (IWGDF) guidelines. In light of the fact that leg ulcer did not stringently adhere to the definition of DFU offered by IWGDF, another post-hoc analyses on population meeting the definition (exclusion of population with leg ulcer) were performed for primary and secondary efficacy endpoints. Also, the post-hoc analysis was done on population based on ABI. Point estimate and confidence intervals of 95% were shown for all efficacy endpoints. Last observation carrying forward (LOCF) was employed for imputing missing data. No multiplicity was performed in the study. All statistical analyses will be conducted using SAS statistical software version 9.4 or higher. All statistical methods in post-hoc analysis were virtually the same to these utilized in main study analysis.

### Role of the funding source

The trial was sponsored by Energenesis Biomedical Co., Ltd. The sponsor was involved in the study design, and had no role in collection, analysis, and interpretation of the data. The manuscript was drafted by the sponsor, but subsequent revisions were made by all authors. All authors had full access to the full data in the study and made the final decision to submit for publication.

## Results

### Demographic and basic characteristics

**Figure 1:**
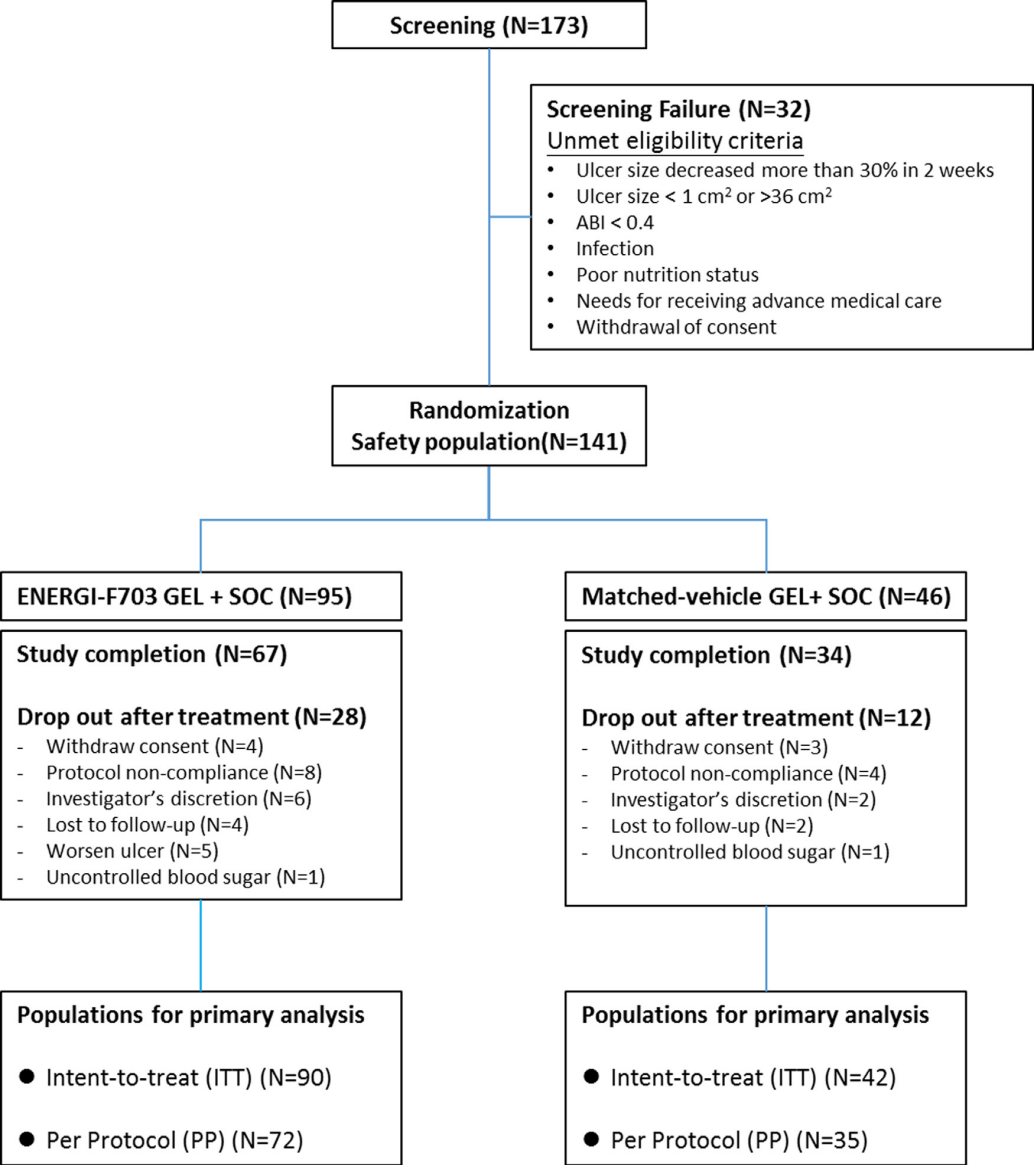
Consolidated Standard of Reporting Trials (CONSORT) flow diagram of the study.

Starting from March 15^th^, 2017 to December 26^th^, 2019, 173 candidates were screened, of which 32 were rejected according to inclusion and exclusion criteria. The remaining 141 eligible patients (81.5%, termed as safety population) were randomized, 95 to the ENERGI-F703 Gel group and 46 to the vehicle gel group. Of these randomized patients, 132 were included in the ITT population (90 patients in the ENERGI-F703 Gel group and 42 in the vehicle gel group) and 107 in the PP population (72 in the ENERGI-F703 Gel group and 35 in the vehicle gel group) according to adherence and additional treatments received (Figure 1).

Randomized patients were also stratified according to target ulcer size (< 16 cm^2^ or ≥ 16 cm^2^) at baseline. The proportions allocated to the ENERGI-F703 Gel and vehicle gel groups were similar and met the expectation of protocol design. Demographics and ulcer characteristics for both treatment groups and the overall population are summarized in Table 1 and Supplemental Table 1. Most target ulcers were Grade 2 (63.6%, N = 84) and located mainly on the foot sole (30.3%, N = 40) or toe (27.3%, N = 36).

**Table 1:** Demographic and baseline characteristics of the randomized patients according to treatment group in the ITT population.

	ENERGI-F703	Vehicle	Total
**ITT Population**, n	90	42	132
**Ethnic origin** (n, %)			
Asian	90 (68.2)	42(31.8)	132 (100)
**Age (years)**			
Mean (SD)	62.0 (14.24)	64.0 (13.38)	62.6 (13.95)
**Gender** (n, %)			
Male	55 (61.1)	26 (61.9)	81 (61.4)
Female	35 (38.9)	16 (38.1)	51 (38.6)
**Body Weight (kg)**			
Mean (SD)	70.9 (16.68)	72.6 (15.43)	71.4 (16.24)
**Body Height (cm)**			
Mean (SD)	162.3 (10.80)	164.4 (9.16)	163.0 (10.32)
**BMI (kg/m^2^)**			
Mean (SD)	26.67 (4.435)	26.78 (4.508)	26.70 (4.441)
**SBP at Ankle**			
Mean (SD)	152.28 (38.213)	158.73 (40.031)	154.25 (38.610)
**SBP at Arms**			
Mean (SD)	139.98 (22.216)	142.64 (19.380)	140.79 (21.289)
**Ankle-Brachial Index**			
Mean (SD)	1.06 (0.241)	1.08 (0.234)	1.07 (0.238)
**DM Duration (years)**			
Median (IQR)	9.59 (11.63)	10.52 (13.17)	9.67 (14.25)
**Current episode duration of DM foot ulcer (days)**			
Median (IQR)	86.00 (125.00)	113.50 (163.00)	100.00 (146.00)
**Baseline Target Ulcer Size (cm^2^)**			
Median (IQR)	3.78 (6.92)	3.47 (7.26)	3.67 (7.00)
Min ∼ Max	1.02 ∼ 38.94	1.03 ∼ 30.40	1.02 ∼ 38.94
**Grade of Foot Ulcers**			
Grade 1	3 (3.3%)	0 (0.0%)	3 (2.3%)
Grade 2	54 (60.0%)	30 (71.4%)	84 (63.6%)
Grade 3	32 (35.6%)	12 (28.6%)	44 (33.3%)
Grade 4	1 (1.1%)	0 (0.0%)	1 (0.8%)
**Target Ulcer Location**			
Ankle	10 (11.1%)	4 (9.5%)	14 (10.6%)
Foot Instep	14 (15.6%)	4 (9.5%)	18 (13.6%)
Foot Sole	26 (28.9%)	14 (33.3%)	40 (30.3%)
Lower Leg	17 (18.9%)	7 (16.7%)	24 (18.2%)
Toe	23 (25.6%)	13 (31.0%)	36 (27.3%)

No statistically significant differences between study groups were observed.

^(1)^SD: Standard deviation; IQR: Interquartile range.

### Drug administration

The mean daily dose frequency was 2 doses per day in both treatment groups. The mean total IP received was 141.7 times (±52.49) in ENERGI-F703 Gel group and 148.3 times (±47.96) in vehicle gel group. The mean treatment duration was 69.8 days (±23.36) and 73.3 days (±22.94) in ENERGI-F703 Gel and vehicle gel group, respectively. Data of drug administration, adherence and exposure was summarized in Table 2.

**Table 2:** Study drug administration, adherence and exposure according to treatment group in the ITT population.

	ENERGI-F703	Vehicle	Treatment Difference
**ITT Population**, n	90	42	
**Total IP Administration [times]**			
Mean (SD)	141.7 (52.49)	148.3 (47.96)	-6.6 (51.10) (95% CI, -25.48∼12.30)
**Treatment Duration [days]**			
Mean (SD)	69.8 (23.36)	73.3 (22.94)	-3.5 (23.22) (95% CI, -12.11∼5.06)
**Treatment Adherence[%]**			
Mean (SD)	101.3 (14.60)	100.7 (5.87)	0.6 (95% CI, -2.95∼4.11)
**Mean Daily Dose [times/day]**			
Mean (SD)	2.0 (0.29)	2.0 (0.12)	0.0 (95% CI, (-0.06∼0.08)

^(1)^SD: Standard deviation; CI: confidence interval.

### Efficacy evaluation of ENERGI-F703 Gel

The primary endpoint of this study was ulcer closure rate in ITT population. A patient with confirmed complete target ulcer closure before the presumed time was counted as a responder. The results showed that the healing response rate was 36.7% and 26.2% for patients receiving ENERGI-F703 Gel and vehicle gel, respectively, with a difference of 9.74% (95% CI = -6.74% – 26.23%) (Table 3). The closure rates were also evaluated in PP population where 44.4% patients receiving ENERGI-F703 Gel and 28.6% patients receiving vehicle gel had reached the ulcer closure during the study (difference: 13.87%, 95% CI = -5.05% – 32.79%) (Supplemental Table 2). Representative photographs of diabetic foot and leg ulcers from patients in ENERGI-F703 Gel treated group are shown in Supplemental Figure 1.

**Table 3:** Complete ulcer closure rate according to treatment group in the ITT population

	ENERGI-F703	Vehicle	Treatment Difference
**Primary outcome** **All Subject**			
ITT, % (n)	36.7% (33)	26.2% (11)	9.74% (95% CI, -6.74% ∼ 26.23%)
**ABI ≤ 1.4**			
ITT, % (n)	36.9% (31)	26.8% (11)	9.43%
			(95% CI, -7.46% ∼ 26.31%)
**ABI > 1.4**			
ITT, % (n)	20.0% (1)	0.0% (0)	20.00%
			(95% CI, -15.06% ∼ 55.06%)
**Foot Ulcers**			
ITT, % (n)	35.6% (26)	25.7% (9)	9.43% (95% CI, -8.73% ∼ 27.59%)
**Leg Ulcers**			
ITT, % (n)	41.2% (7)	28.6% (2)	10.34% (95% CI, -30.67% ∼ 51.36%)
**Baseline Ulcer Grade = 1**			
ITT, % (n)	33.3% (1)		
**1.5 cm^2^ ≤ Ulcer Size < 25 cm^2^**			
ITT, % (n)	33.3% (1)		
**Baseline Ulcer Grade = 2**			
ITT, % (n)	40.7% (24)	17.2% (5)	19.99% (95% CI, 0.64% ∼ 39.34%)
**1.5 cm^2^ ≤ Ulcer Size < 25 cm^2^**			
ITT, % (n)	36.7% (18)	9.1% (2)	27.64% (95% CI, 9.57% ∼ 45.71%)
**Baseline Ulcer Grade ≤ 2**			
ITT, % (n)	40.3% (25)	17.2% (5)	19.63% (95% CI, 0.49% ∼ 38.78%)
**1.5 cm^2^ ≤ Ulcer Size < 25 cm^2^**			
ITT, % (n)	36.5% (19)	9.1% (2)	27.45% (95% CI, 9.68% ∼ 45.21%)
**Baseline Ulcer Grade = 3**			
ITT, % (n)	28.6% (8)	38.5% (5)	-9.89% (95% CI, -41.19% ∼ 21.41%)
**1.5 cm^2^ ≤ Ulcer Size < 25 cm^2^**			
ITT, % (n)	29.2% (7)	36.4% (4)	-7.20% (95% CI, -40.94% ∼ 26.55%)

^(1)^CI = confidence interval.

^(2)^ABI = ankle brachial index.

^(3)^ABI of a subject in ENERGI-F703 treatment group was unknown and excluded from the results.

Peripheral arterial disease (PAD) occurs commonly among persons with diabetic foot and leg ulcers. PAD impedes wound healing and increase the risk of LEA. To investigate the effect of PAD in ENERGI-F703 Gel efficacy, the primary endpoint was analyzed by stratifying patients into two subgroups of ABI ≤ 1.4 and ABI > 1.4.

In the ITT patients with ABI ≤ 1.4, the healing response rate was 36.9% in the ENERGI-F703 Gel group and 26.8% in the vehicle gel group (difference = 9.43%, 95% CI = -7.46% – 26.31%) (Table 3). Similarly in the PP population, patients with ABI ≤ 1.4 receiving ENERGI-F703 Gel showed a higher response rate than patients receiving vehicle gel (43.5% vs. 29.4%, difference = 12.03%, 95% CI = -7.29% – 31.34%) (Supplemental Table 2). For ITT patients of ABI > 1.4, there were 5 patients in the ENERGI-F703 Gel group and 1 patient in the vehicle gel group (Table 3). The number of the PP patients with ABI > 1.4 was two in the ENERGI-F703 Gel group and one in the vehicle gel group (Supplemental Table 2). The sample size of the patients with ABI > 1.4 is too small to effectively test the difference between the ENERGI-F703 Gel and the vehicle gel group.

To explore the effect of the foot and leg ulcers in ENERGI-F703 Gel efficacy, the primary endpoint was analyzed by layering patients into two subgroups of foot and leg ulcers. In the ITT patients with foot ulcers, the healing response rate was 35.6% in the ENERGI-F703 Gel group and 25.7% in the vehicle gel group (difference = 9.43%, 95% CI = -8.73% – 27.59%) (Table 3). Similarly in the PP population, patients with foot ulcers receiving ENERGI-F703 Gel showed a higher response rate than patients receiving vehicle gel (43.1% vs. 30.0%, difference = 11.72%, 95% CI = -8.99% – 32.44%) (Supplemental Table 2). For ITT patients of leg ulcers, there were 17 patients in the ENERGI-F703 Gel group and 7 patients in the vehicle gel group (Table 3). The number of the PP patients with leg ulcer was 14 in the ENERGI-F703 Gel group and 5 in the vehicle gel group (Supplemental Table 2). Considering the small sample size of the patients with leg ulcers, the results may not be representative of the population.

To identify the population demonstrating greatest ENERGI-F703 Gel efficacy, the primary endpoint was further analyzed by sub-grouping patients according to baseline ulcer Grade and size. In the ITT population, patients with a Grade 1 or 2 target ulcer receiving ENERGI-F703 Gel demonstrated a higher response rate than patients matched for target ulcer severity receiving vehicle gel (40.3% vs. 20.7%, difference = 19.63%, 95% CI = 0.49% – 38.78%) (Table 3). Similarly in the PP population, patients with Grade 1 or 2 target ulcer receiving ENERGI-F703 Gel showed a higher response rate than patients matched for target ulcer severity receiving vehicle gel (48.0% vs. 20.8%, difference = 27.17%, 95% CI = 5.82% – 48.52%) (Supplemental Table 2). Furthermore, among patients with Grade 1 or 2 target ulcers at baseline and ulcer size ≥ 1.5 cm^2^ but < 25 cm^2^, those receiving ENERGI-F703 Gel demonstrated a greater response rate, both for the ITT population (36.5% vs. 9.1%, difference = 27.45%, 95% CI = 9.68% – 45.21%) and PP population (43.9% vs. 10.0%, difference = 33.9%, 95% CI = 13.81% – 53.99%) (Table 3 and Supplemental Table 2). Similar results were observed when 24 patients with the target ulcer at lower leg (17 patients in ENERGI-F703 Gel vs. 7 patients in vehicle gel) were excluded from the analyses (Supplemental Table 3).

Time to confirmed complete ulcer closure was evaluated and the median for either treatment group could not be obtained by the last observation timepoint (12 weeks). 25% quartiles (Q1) of time to confirmed complete ulcer closure was 69 days (95% CI = 53.0 – 84.0) in the ENERGI-F703 Gel group and 84 days (95% CI = 70.0 –) in the vehicle gel group (Table 4). For patients with Grade 1/2 ulcer at baseline, Q1 time to complete closure was 58 days (95% CI = 51.0 – 84.0) in ENERGI-F703 Gel group and > 12 weeks (95% CI = 56.0 –) in vehicle gel group in ITT population (*p* = 0.0380 by Log-rank test, *p* = 0.0334 by Wilcoxon test, n = 91). Similar results were observed in the PP population (58 days vs > 12 weeks, *p* = 0.0180 by Log-rank test, *p* = 0.0158 by Wilcoxon test, n = 74, Supplemental Table 4).

**Table 4:** Time to complete ulcer closure according to treatment group in the ITT population

	ENERGI-F703	Vehicle	*P*-ValueLOGRANK	*P*-ValueWILCOXON
**All Subject**				
ITT, n Q1 days (95% CI)	90 69 (53.0 ∼ 84.0)	42 84 (70.0 ∼)	0.1754	0.1526
**ABI ≤ 1.4**				
ITT, n	84	41	0.2045	0.1643
Q1 days (95% CI)	67 (52.0 ∼ 84.0)	84 (70.0 ∼)		
**ABI > 1.4**				
ITT, n	5	1	0.5637	0.5637
Q1 days (95% CI)	84 (84.0 ∼)	-		
**Foot Ulcers**				
ITT, n Q1 days (95% CI)	73 67 (51.0 ∼ 84.0)	35 84 (70.0 ∼)	0.1770	0.1110
**Leg Ulcers**				
ITT, n Q1 days (95% CI)	17 69 (27.0 ∼ 87.0)	7 70 (56.0 ∼)	0.7449	0.9546
**Baseline Ulcer Grade ≤ 2**				
ITT, n Q1 days (95% CI)	62 58 (51.0 ∼ 84.0)	29 - (56.0 ∼)	0.0380	0.0334
**1.5 cm^2^ ≤ Ulcer Size < 25 cm^2^**				
ITT, n Q1 days (95% CI)	52 67 (51.0 ∼ 87.0)	22 - (70.0 ∼)	0.0091	0.0090
**Baseline Ulcer Grade = 3**				
ITT, n Q1 days (95% CI)	28 84 (28.0 ∼)	13 70 (39.0 ∼)	0.6567	0.6464
**1.5 cm^2^ ≤ Ulcer Size < 25 cm^2^**				
ITT, n Q1 days (95% CI)	24 84 (42.0 ∼)	11 70 (39.0 ∼)	0.7188	0.6448

^(1)^ ABI = ankle brachial index.

^(2)^ ABI of a subject in ENERGI-F703 treatment group was unknown and excluded from the results.

^(3)^ “-“ = not able to estimate Q1 days.

**Figure 2:**
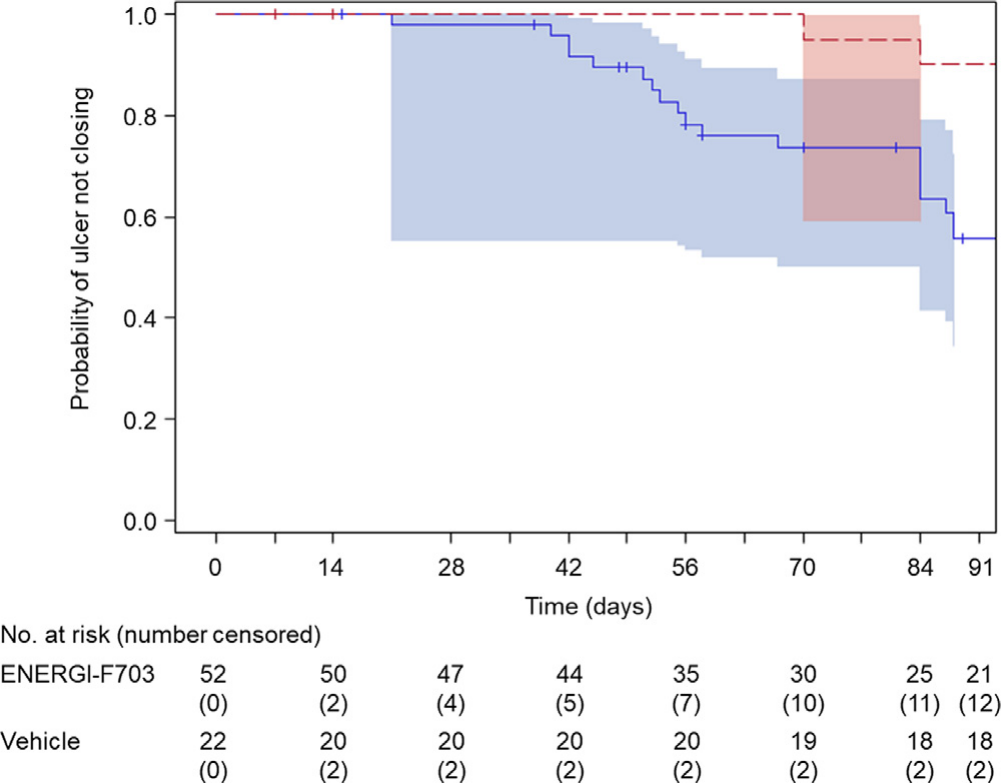
**Kaplan-Meier with CI diagram of time to complete ulcer closure with ENERGI-F703 Gel compared to vehicle gel for patients with baseline ulcer ≥ 1.5 cm^2^ but < 25 cm^2^, those with ≤ Grade 2 ulcer at baseline in the ITT population.** The comparison between two groups was performed by log-rank test. The ENERGI-F703 Gel group showed an increased cumulative complete ulcer closure rate than vehicle gel group.

Furthermore, for ITT patients with baseline ulcer ≥ 1.5 cm^2^ but < 25 cm^2^, those with ≤ Grade 2 ulcer at baseline receiving ENERGI-F703 Gel demonstrate Q1 time to closure of 67 days (95% CI = 51.0 – 87.0) and >12 weeks for vehicle gel group (95% CI = 70.0 –) (*p* = 0.0091 by Log-rank test, *p* = 0.0090 by Wilcoxon test, n = 74) (Table 4). Similar results were observed in the PP population (67 days vs > 12 weeks, *p* = 0.0080 by Log-rank test, *p* = 0.0078 by Wilcoxon test, n = 61, Supplemental Table 4). For this subgroup, the overall cumulative distributions of time to complete target ulcer closure were analyzed by constructing Kaplan-Meier curves (Figure 2). Similar results were observed when patients with the target ulcer at lower leg were excluded from the analyses (Supplemental Table 5). It is noticed that the same conclusion was made by either Log-rank test or Wilcoxon test.

With regard to ABI, the ITT patients with ABI ≤ 1.4 had Q1 of time to confirmed complete ulcer closure of 67 days (95% CI = 52.0 – 84.0) in the ENERGI-F703 Gel group and 84 days (95% CI = 70.0 –) in the vehicle gel group (*p* = 0.2045 by Log-rank test, *p* = 0.1643 by Wilcoxon test, n = 125) (Table 4). Similar results were observed in the PP population. The Q1 time to complete closure of PP patients with ABI ≤ 1.4 was 67 days (95% CI = 45.0 - 84.0) in ENERGI-F703 Gel group and 84 days (95% CI = 70.0 –) in vehicle gel group (*p* = 0.1446 by Log-rank test, *p* = 0.1172 by Wilcoxon test, n = 103) (Supplemental Table 4). For ITT and PP patients of ABI > 1.4, the sample size is too small to test the difference between the ENERGI-F703 Gel group and the vehicle gel group.

As for the foot and leg ulcers, the ITT patients with foot ulcers had Q1 of time to confirmed complete ulcer closure of 67 days (95% CI = 51.0 – 84.0) in the ENERGI-F703 Gel group and 84 days (95% CI = 70.0 –) in the vehicle gel group (*p* = 0.1770 by Log-rank test, *p* = 0.1110 by Wilcoxon test, n = 108) (Table 4). Similar results were observed in the PP population. The Q1 time to complete closure of PP patients with foot ulcers was 67 days (95% CI = 45.0 - 84.0) in ENERGI-F703 Gel group and 84 days (95% CI = 70.0 –) in vehicle gel group (*p* = 0.1704 by Log-rank test, *p* = 0.1102 by Wilcoxon test, n = 88) (Supplemental Table 4). For ITT and PP patients of leg ulcers, the small sample size could not test the difference between the ENERGI-F703 Gel group and the vehicle gel group.

### Safety evaluation

The adverse events (AEs) were coded using MedDRA dictionary version 20.0. Incidence rates of AEs are summarized in Table 5. Among the 141 patients included in the safety analysis (the safety population), 99 (70.2%) experienced at least one treatment emergent adverse event, of which 14 (9.9%) were treatment-related AEs. The most common treatment-related AEs were in the System Organ Class (SOC) “general disorders and administration site conditions” (11 patients, 7.8%) and “administration site pain” was the main condition encountered. “Administration site pain” was the only event with incident rate more than 5%. Besides, there were 5 patients (5.3%) in ENERGI-F703 Gel group and 2 patients (4.3%) in vehicle gel group experiencing target ulcer related AEs of infection and infestation. In addition, 39 patients (27.7%) experienced at least one serious adverse event (SAE), including 5 (3.5%) fatalities; no SAEs were judged as treatment-related. Two most frequent causes of SAEs were infection and cardiac disorder. However, incidences of all AEs and SAEs did not differ between treatment groups. Furthermore, no suspected unexpected serious adverse reactions (SUSARs) were reported throughout the study. In addition to AEs, the safety assessment included vital signs, physical examinations, 12-Lead ECG, and laboratory tests. At the end of the study, the values of glucose, HbA1c, BUN and creatinine were higher than baseline in both treatment groups, but only the difference of glucose value showed statistical significance (mean increase of 9.13 and 33.26 mg/dL in the ENERGI-F703 Gel and Vehicle gel group, *p* = 0.0041; LsMean 13.91 vs. 63.74, difference = -49.83, 95% CI of LsMean = -90.312 – -9.354).

**Table 5:** Summary of adverse events on safety population according to treatment group

	ENERGI-F703	Vehicle	Total
Safety Population, n	95	46	141
Subjects with at least one treatment emergent adverse event (AE), n (%)	65 (68.4%)	34 (73.9%)	99 (70.2%)
Subject with target ulcer related adverse events (AEs), n (%)	22 (23.2%)	11 (23.9%)	33 (23.4%)
Subjects with Grade ≥3 adverse events (AEs), n (%)	19 (20.0%)	10 (21.7%)	29 (20.6%)
Subjects with treatment related adverse events (AEs), n (%)	9 (9.5%)	5 (10.9%)	14 (9.9%)
Subject with treatment modified adverse events (AEs), n (%)	6 (6.3%)	3 (6.5%)	9 (6.4%)
Subjects with serious adverse events (SAEs), n (%)	27 (28.4%)	12 (26.1%)	39 (27.7%)
Subjects with suspected unexpected serious adverse reactions (SUSARs), n (%)	0 (0.0%)	0 (0.0%)	0 (0.0%)
Subjects with target ulcer related infections and infestations, n (%)	5 (5.3%)	2 (4.3%)	7 (5.0%)
Subjects with frequency of adverse events (AEs) ≥ 5%, n (%)			
Administration site pain	6 (6.3%)	7 (15.2%)	13 (9.2%)

## Discussion

The randomized, double-blind, vehicle-controlled, parallel, multicentre, phase II study evaluated the efficacy and safety of ENERGI-F703 Gel in patients with diabetic foot and leg ulcers. ENERGI-F703 Gel was considered as a safe and well-tolerated treatment for chronic diabetic foot and leg ulcers. No SUSARs related to ENERGI-F703 Gel were reported throughout the study, although many patients experienced various AEs or SAEs during the study period. The relatively high incidences of AEs and SAEs can be explained by the inclusion of many elderly DM patients with multiple chronic comorbidities. However, most patients who experienced SAEs recovered or were stable by the end of this study except for 5 death counts occurring during the study. None of these SAEs were deemed treatment-related by investigators. “Administration site pain” was the most common treatment-related AE, reported by ten patients from three study centers of which eight originated from the same study center. Nonetheless, we found no statistically significant difference in the incidences of “administration site pain” between treatment groups. Accordingly, “administration site pain” could be reasonably considered incidental and study center-specific, and should not limit the clinical application of ENERGI-F703 Gel.

Patients were also monitored for basic laboratory health metrics during the study. The most common abnormal values with clinical significance were related to DM. At baseline, both treatment groups exhibited mean glucose levels, mean HbA1c levels, mean creatinine values, and mean blood urea nitrogen values outside the normal health range, reflecting long-standing diabetes, and chronic kidney diseases complicated by diabetes. Patients with diabetes are also prone to infection so white blood cell counts may appear clinically abnormal at times. At the end of treatment, there was slight worsening of laboratory values related to diabetes and renal function were observed, but these values did not differ statistically significantly between treatment groups, indicating that these changes were attributable to disease progression rather than effects of the intervention. However, the increased in fasting glucose was statistically significantly smaller in the ENERGI-F703 Gel group than the vehicle gel group (9.13 vs. 33.26 mg/dL, *p* = 0.0041). The clinical relevance of this difference warrants further study.

The Wagner ulcer classification system assesses DFUs based on the depth of penetration, presence of osteomyelitis or gangrene, and extent of tissue necrosis. The DFUs with higher Wagner Grade, therefore, are thought to be more difficult to heal. However, the Wagner ulcer classification system is not appropriate for assessing diabetic leg infections. In agreement with this notion, those stratified by ulcer Grade at baseline with Grade 2 diabetic foot and leg ulcers showed a better response rate than those with Grade 3 (40.3% vs 30%) among patients treated with ENERGI-F703 Gel. However, we found that the healing rate of patients with Grade 3 lesions (38%) was better than those with Grade 2 (20%) among patients treated with vehicle gel, contradicting the above notion. Upon closer inspection, we found that patients whose ulcers were located at the toe contributed to the high response rate. Compared to other locations of the foot, the toes have much less tissue between the skin and bone. Therefore, the lesions that might be considered Grade 2 at other areas of foot according to the depth of penetration might appear to be more severe (Grade 3) at the toe as defined by the Wagner grading system, a potential pitfall of an otherwise standardized method. Three out of four patients in the vehicle gel group with Grade 3 lesions located at the toe showed an apparently high treatment response rate (75%), whereas only two out of nine patients with lesions elsewhere showed a response rate of 22%. No patients with Grade 3 lesions located at the toe were in ENERGI-F703 Gel group. Accordingly, even distribution of ulcer locations between the two study groups should be considered in the next study.

The causes of diabetes foot ulcers are multifactorial and many factors, including wound depth and location, blood glucose control, the health of peripheral circulation, and patient lifestyle can influence treatment responses. As phase II trials aim mainly to provide proof of treatment principle, identify parameters influencing efficacy, and evaluate treatment safety, preferably in a heterogeneous population, relatively broad criteria were set for patient enrolment, including enlarging the ulcer size limit (36 cm^2^) and ulcer Grade (Wager Grade 3) and lowering health requirements for the peripheral vascular (ABI > 0.4). The results revealed statistically significantly greater efficacy in healing diabetic foot and leg ulcers of Grade ≤ 2 and >1.5 cm^2^, while ulcers smaller than 1.5 cm^2^ in size healed relatively well in both ENERGI-F703 Gel and vehicle gel groups (70.0% and 57.1%) with difference of 12.86% (95 % CI = -33.52% - 59.23%). Regarding ABI and the location of ulcers, no statistically significant difference of the efficacy between the ENERGI-F703 Gel and Vehicle gel group was noted. Even though ENERGI-F703 Gel failed to substantially improve complete healing (the primary efficacy endpoint), results strongly support that potential of topical adenine for diabetic foot and leg ulcers treatment. Thus, ENERGI-F703 Gel should be considered for phase III trials

Effective therapeutic intervention for diabetic foot and leg ulcers has proven challenging due to the variety of risk factors. In this study, we provide clinical evidence from a randomized vehicle-controlled trial that a topical adenine-containing gel can safely accelerate diabetic foot and leg ulcers healing, presumably by increasing the cellular energy required for tissue repair and regeneration.

Supplementation of oxygen and nutrient delivery from the blood circulation can promote tissue healing. Maintaining cellular ATP is essential for every aspect of the wound healing process.^19^ However, ATP is unstable at room temperature and cell membrane impermeable, so application of the unmodified form is unfeasible for wound treatment. Alternatively, multiple studies have revealed that the intracellular delivery of ATP via fusogenic lipid vesicles (ATP-Vesicles) can statistically significantly enhance skin wound healing in rodents^12^ and rabbits.^13^^,^^14^ In fact, ATP-Vesicles provided greater benefit than Regranex®, the only FDA-approved prescription drug for diabetes wounds.^20^ Another strategy is application of cell-permeable ATP precursors; indeed, extracellular adenine can increase intracellular ATP level around 1.5-fold in several cell types^15^ and also statistically significantly shortened the time to full-thickness wound healing in a diabetes mouse model.^16^ This efficacy is due to the efficiency with which adenine is transported into cells via equilibrative nucleoside transporters (ENTs)^21^, ^22^, ^23^ and the stability of this compound under various storage conditions, in contrast to ATP. The pattern of healing in ATP-enriched wounds is totally different from conventional healing in which the wound bed is first covered by a provisional matrix, followed by the appearance of granulation tissue after 3‒6 days. By contrast, ATP-enriched wounds show faster healing without hypertrophic scar formation,^13^^,^^20^ possibly due to the activation of macrophages within 24 h during the granulation stage, which subsequently enhance collagen production and neovascularization.^24^ Accordingly, increasing the intracellular energy (ATP) is a novel method to induce a unique and efficient healing process. Here, we provide the first proof for the efficacy of this strategy in human patients. Based on the far-reaching effects of ATP increment exerted by ENERGI-F703 Gel, future prospective applications would be expanded to the treatment to different types of disorders like chronic wound or deep wound with infection.

This study still has several limitations. The 12-week treatment duration may have been too brief to assess the effect on DFUs ≥ Grade 3 wounds. Further, efficacy evaluation was limited by the Wagner grading system, which requires improvement, especially for toe lesions. It is also difficult to accurately assess diabetic leg ulcers and the surface area of deeper ulcers. No planned offloading implementation to relieve the pressure and enhance wound healing was another limitation. The relevant diabetic wound classification system,^25^, ^26^, ^27^ detail neuropathic assessment, standardized offloading, and longer follow up will be adopted in future trial designs. We expect to re-evaluate the efficacy of ENERGI-F703 Gel for treating Grade ≥ 3 wounds with a more relevant grading method, measurement of neuropathy, offloading implementation, and longer follow up. Despite these limitations, this study provides support for a novel therapeutic concept that healing can be accelerated by maintaining cellular energy charge at the wound site.

In conclusion, our results suggest that adenine-containing ENERGI-F703 Gel is safe and can promote the closure of diabetic foot and leg ulcers. In order to get more reliable results, more stringent criteria will also be set, including more precise ulcer location for appropriate classification, more detailed standard of care design, consistent offloading implementation, measurement of neuropathy and factoring in the use of treatment for arterial insufficiency and its potential impact on the results. Patient sample size will be carefully determined through statistical method to reach a conclusion with statistical validation. In the next stage, the efficacy of ENERGI-F703 Gel will be further confirmed in a larger population with more precise stratification for ulcer size and ulcer Grade. Future research will focus on more precise target populations and longer treatment period for the optimization of treatment scheme and clearer demonstration of safety profile.

## Contributors

JY Yang, CC Chen, SC Chang, JT Yeh, and HF Huang contributed equally to this work. The study protocol was approved by JY Yang, CC Chen, SC Chang, HF Huang and NT Dai. JY Yang, CC Chen, SC Chang, JT Yeh, HF Huang, HC Lin, SH Lin, YH Lin, LG Wei, Tom J. Liu, SY Hung, HM Yang, HH Chang, CH Wang, YS Tzeng, CH Huang, CY Chou, YS Lin, SY Yang were the eight medical centers coordinating investigators of the study and formed the trial steering committee with NT Dai. HM Chen, JT Lin, YF Cheng, and YC Kuo did the protocol design and literature search. The report was written by JT Lin, YF Cheng, GH Young, and CF Huang, and reviewed by the trial steering committee, who took responsibility for the conduct of the trial, the integrity of the data and the overall content of the report. All authors approved the final submitted version of the report and decided to submit for publication.

## Data sharing statement

All data requests should be submitted to the corresponding author for consideration. After signing Data Access Agreement, the data will be shared through a secure online platform for a limited duration.

## Declaration of interests

This project was sponsored by Energenesis Biomedical Co. Ltd. Han-Min Chen, Jiun-Tsai Lin, Yi-Fang Cheng, Guang-Huar Young, Chun-Fang Huang, Ya-Chun Kuo are current employees of Energenesis Biomedical Co., Ltd. All other authors have no conflicts of interest to declare.

## Appendix Supplementary materials

Image, application 1
