# Supplementary material for: ENERGI-F703 gel, as a new topical treatment for diabetic foot and leg ulcers: A multicenter, randomized, double-blind, phase II trial
Source: eClinicalMedicine. 2022 Jul 10;51:101497. doi: 10.1016/j.eclinm.2022.101497 (PMC9284381; doi:10.1016/j.eclinm.2022.101497)
Supplement: Supplementary file 1 [file mmc1.docx]

| **Wound Size** | **Wagner’s Grade** | **(a)** | **(b)** |
| --- | --- | --- | --- |
| 3.34 cm^2^ | 2 | 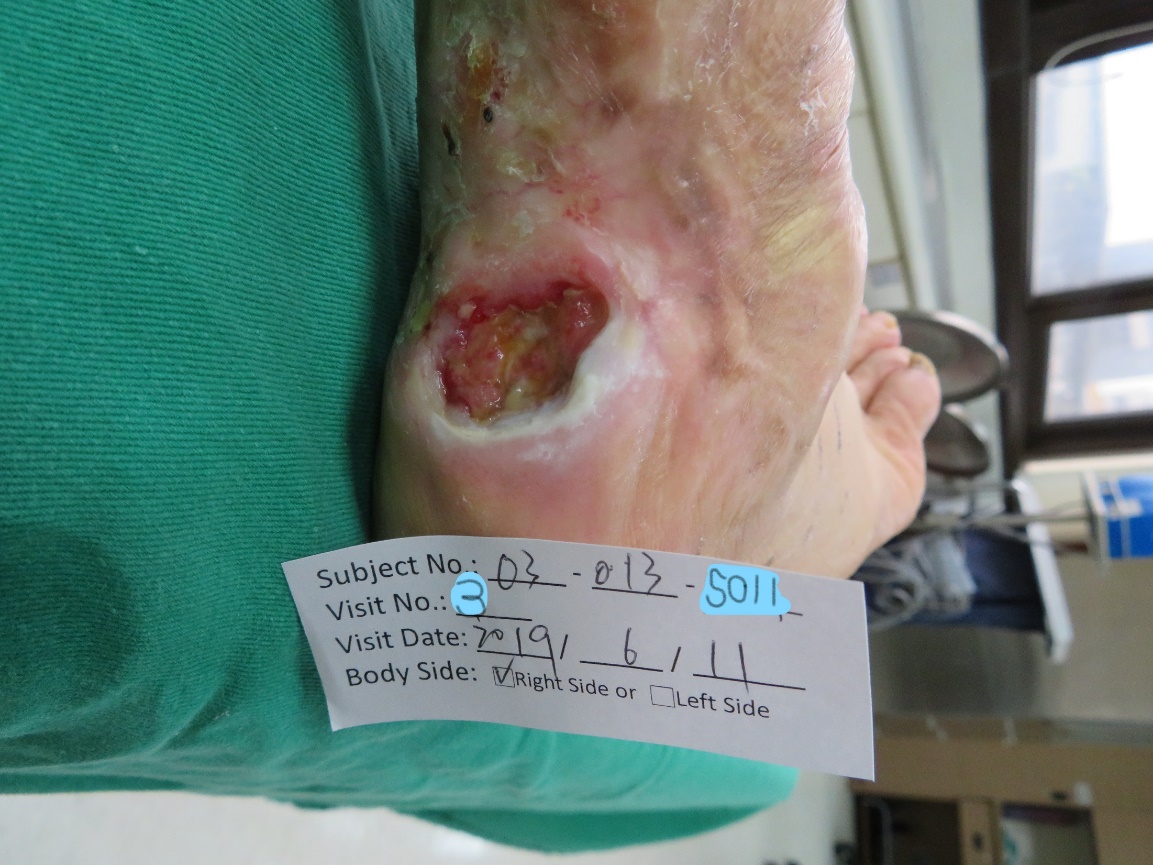 | 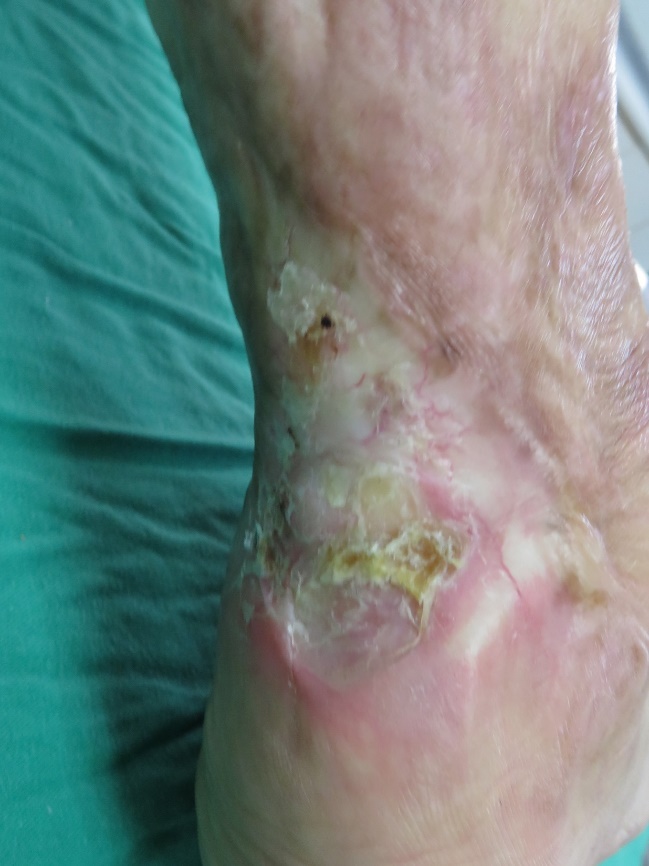 |
| 6.38 cm^2^ | 2 | 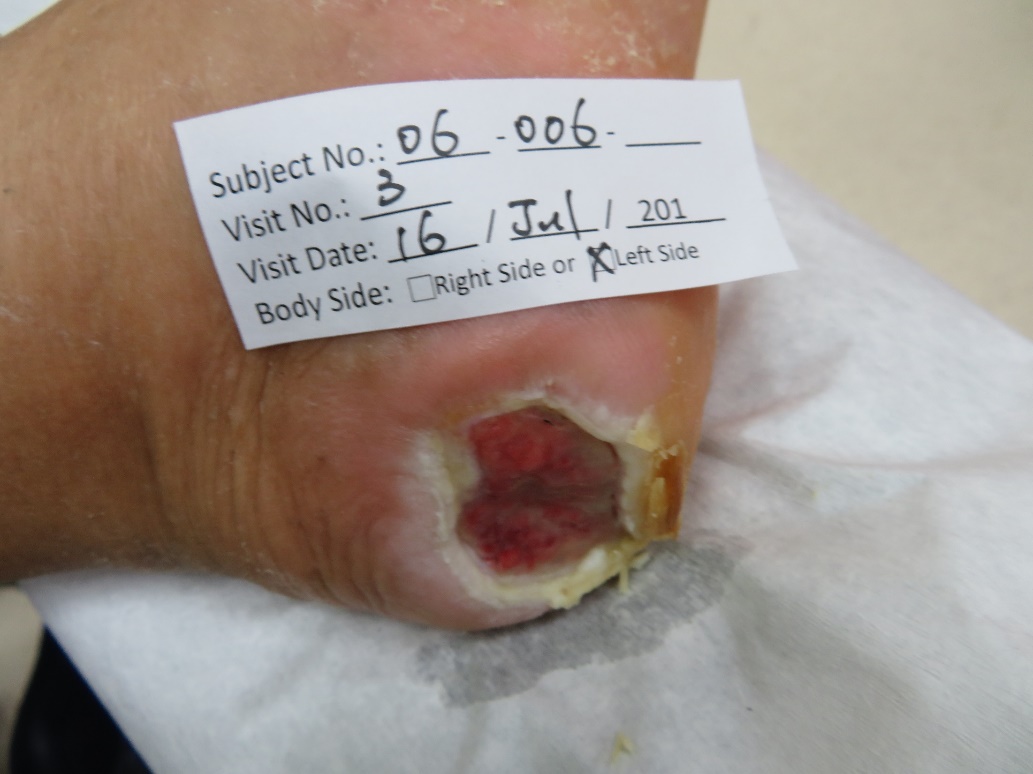 | 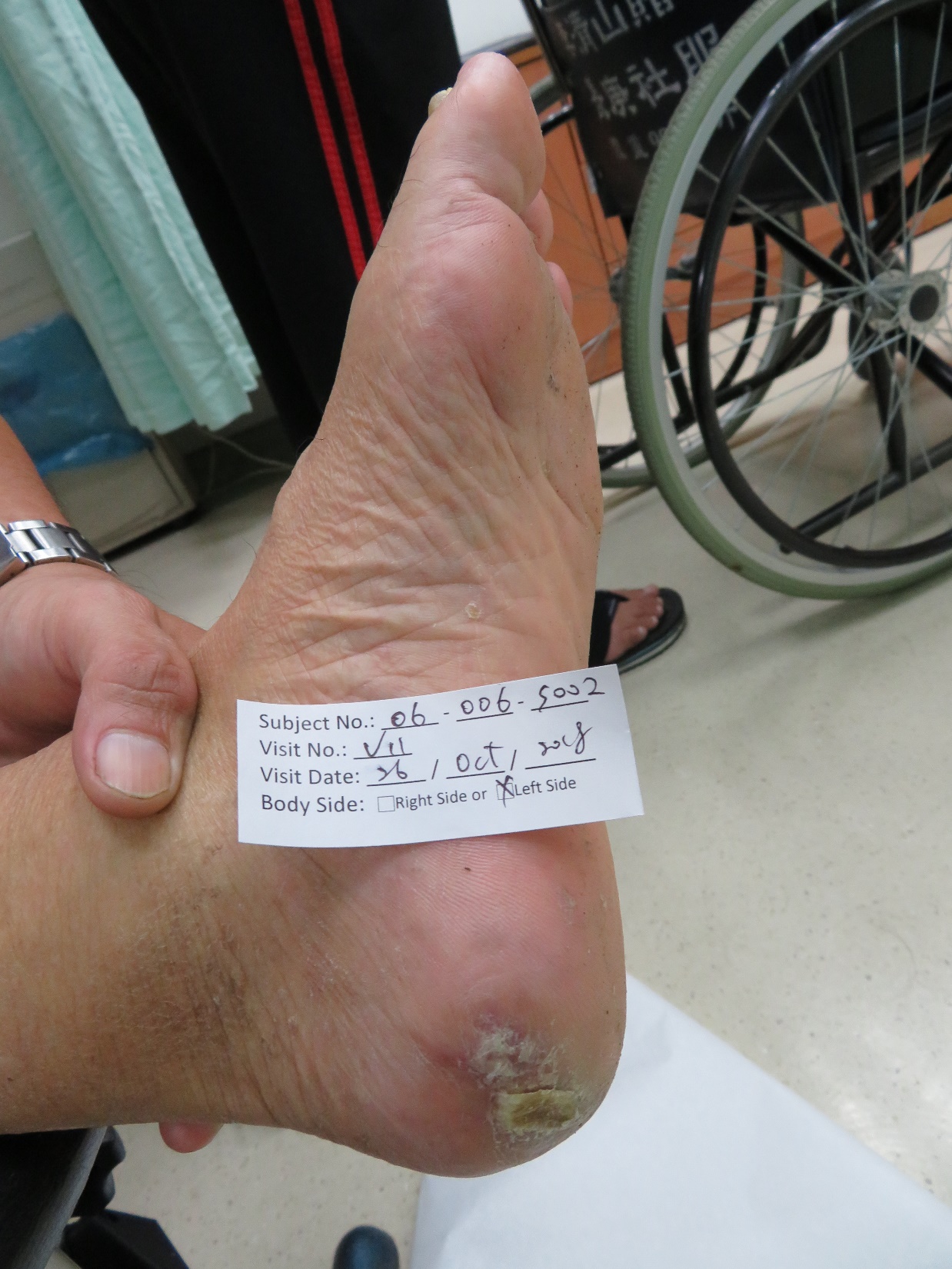 |
| 4.11 cm^2^ | 3 | 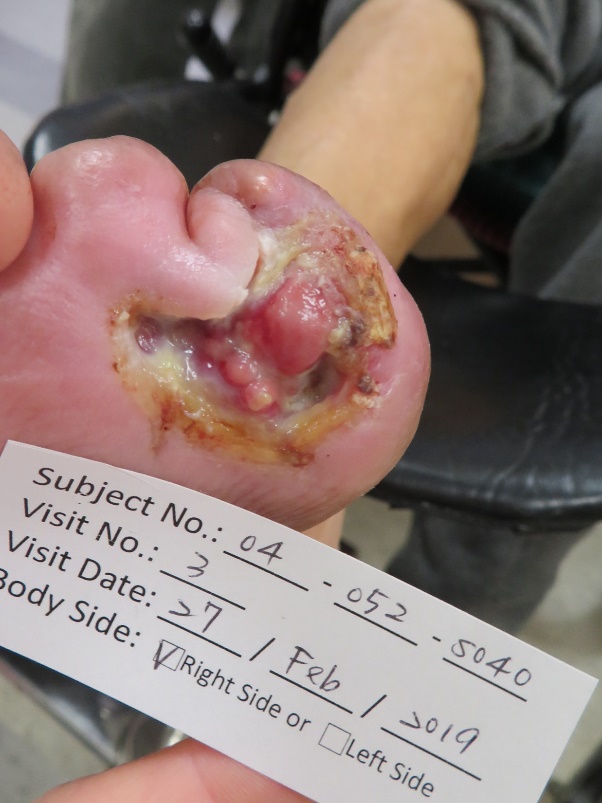 | 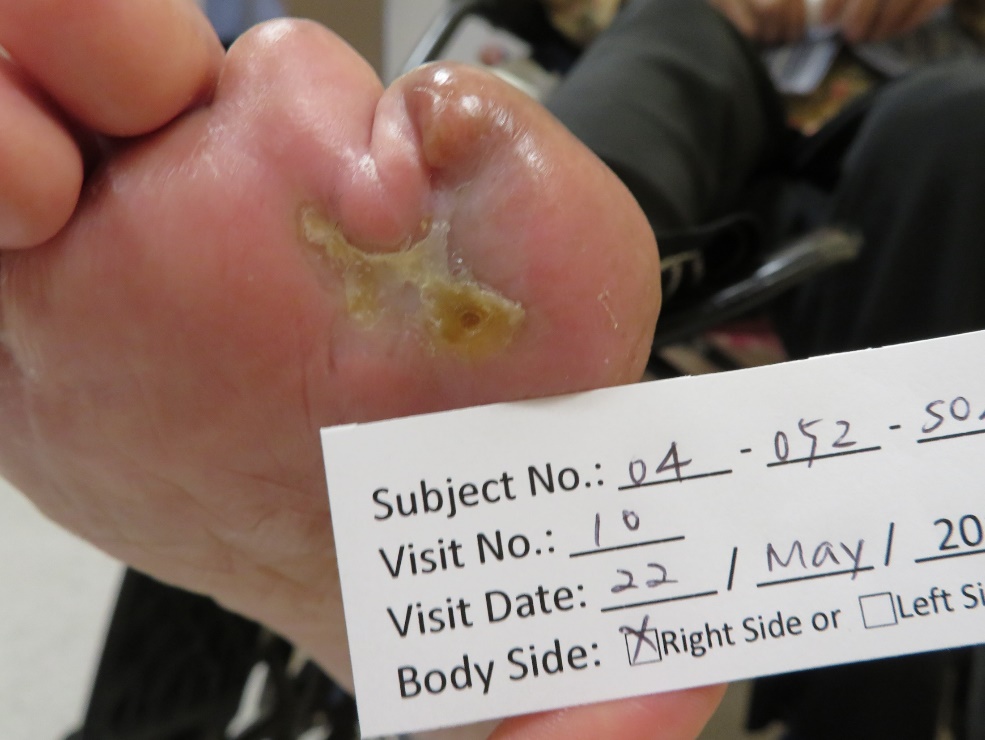 |
| 23.10 cm^2^ | 3 | 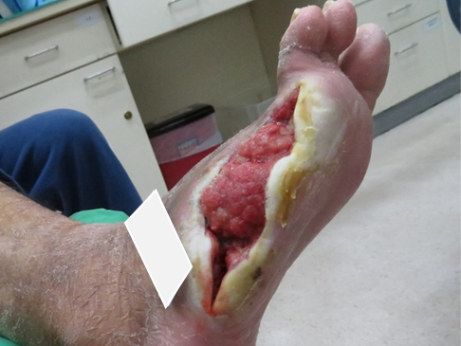 | 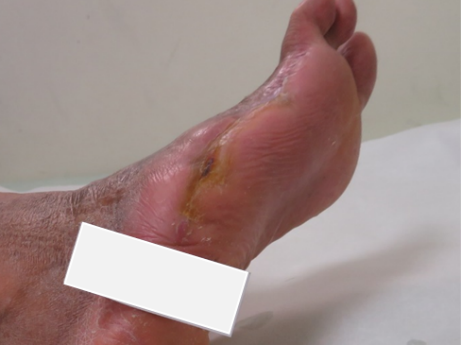 |

**Supplemental Figure 1. The representative photos of the target ulcers of the patients treated with ENERGI-F703 Gel at (a) baseline and (b) healing.**

Supplemental Table 1. Patient recruitment according to study sites

|  | **ENERGI-F703** | **Vehicle** | **Total** |
| --- | --- | --- | --- |
| **ITT Population**, n | 90 | 42 | 132 |
| **Grade of Foot Ulcers** |  |  |  |
| Site 01: TSGH | 14 (15.6%) | 7 (16.7%) | 21 (15.9%) |
| Site 02: NTUH | 5 (5.6%) | 2 (4.8%) | 7 (5.3%) |
| Site 03: CGMF-Taipei | 7 (7.8%) | 2 (4.8%) | 9 (6.8%) |
| Site 04: SKMH | 39 (43.3%) | 20 (47.6%) | 59 (44.7%) |
| Site 05: TYAFGH | 7 (7.8%) | 2 (4.8%) | 9 (6.8%) |
| Site 06: CGMF-Linkou | 6 (6.7%) | 4 (9.5%) | 10 (7.6%) |
| Site 07: NTUH-YL | 9 (10.0%) | 3 (7.1%) | 12 (9.1%) |
| Site 08: SHH | 3 (3.3%) | 2 (4.8%) | 5 (3.8%) |

Supplemental Table 2. Complete ulcer closure rate according to treatment group in the PP population

|  | **ENERGI-F703** | **Vehicle** | **Treatment Difference** |
| --- | --- | --- | --- |
| **Primary outcome**  **All Subject** |  |  |  |
| PP, % (n) | 44.4% (32) | 28.6% (10) | 13.87%  (95% CI, -5.05% ~ 32.79%) |
| **ABI ≤ 1.4** |  |  |  |
| PP, % (n) | 43.5% (30) | 29.4% (10) | 12.03% |
|  |  |  | (95% CI, -7.29% ~ 31.34%) |
| **ABI > 1.4** |  |  |  |
| PP, % (n) | 50.0% (1) | 0.0% (0) | 50.00% |
|  |  |  | (95% CI, -19.30% ~ 100.00%) |
| **Foot Ulcers** |  |  |  |
| PP, % (n) | 43.1% (25) | 30.0% (9) | 11.72%  (95% CI, -8.99% ~ 32.44%) |
| **Leg Ulcers** |  |  |  |
| PP, % (n) | 50.0% (7) | 20.0% (1) | 31.54%  (95% CI, -22.16% ~ 85.24%) |
| **Baseline Ulcer Grade = 1** |  |  |  |
| PP, % (n) | 33.3% (1) |  |  |
| **1.5 cm^2^ ≤ Ulcer Size < 25 cm^2^** |  |  |  |
| PP, % (n) | 33.3% (1) |  |  |
| **Baseline Ulcer Grade = 2** |  |  |  |
| PP, % (n) | 48.9% (23) | 20.8% (5) | 28.10%  (95% CI, 6.46% ~ 49.74%) |
| **1.5 cm^2^ ≤ Ulcer Size < 25 cm^2^** |  |  |  |
| PP, % (n) | 44.7% (17) | 10.0% (2) | 34.74%  (95% CI, 14.17% ~ 55.30%) |
| **Baseline Ulcer Grade ≤ 2** |  |  |  |
| PP, % (n) | 48.0% (24) | 20.8% (5) | 27.17%  (95% CI, 5.82% ~ 48.52%) |
| **1.5 cm^2^ ≤ Ulcer Size < 25 cm^2^** |  |  |  |
| PP, % (n) | 43.9% (18) | 10.0% (2) | 33.90%  (95% CI, 13.81% ~ 53.99%) |
| **Baseline Ulcer Grade = 3** |  |  |  |
| PP, % (n) | 36.4% (8) | 45.5% (5) | -9.09%  (95% CI, -44.73% ~ 26.54%) |
| **1.5 cm^2^ ≤ Ulcer Size < 25 cm^2^** |  |  |  |
| PP, % (n) | 36.8% (7) | 44.4% (4) | -7.60%  (95% CI, -46.65% ~ 31.44%) |

1. CI = confidence interval.
2. ABI = ankle brachial index.
3. ABI of a subject in ENERGI-F703 treatment group was unknown and excluded from the results.

Supplemental Table 3. Complete ulcer closure rate excluding subjects with lower leg ulcer

|  | **ENERGI-F703** | | **Vehicle** | **Treatment Difference** |
| --- | --- | --- | --- | --- |
| **All Subject** |  |  | |  |
| ITT, % (n) | 35.6% (26) | 25.7% (9) | | 9.43% |
|  |  |  | | (95% CI, -8.73%~27.59%) |
| PP, % (n) | 43.1% (25) | 30.0% (9) | | 11.72% |
|  |  |  | | (95% CI, -8.99%~32.44%) |
| **Baseline Ulcer Grade = 1** |  |  | |  |
| ITT, % (n) | 50.0% (1) |  | |  |
| PP, % (n) | 50.0% (1) |  | |  |
| **1.5 cm^2^ ≤ Ulcer Size < 25 cm^2^** |  |  | |  |
| ITT, % (n) | 50.0% (1) |  | |  |
| PP, % (n) | 50.0% (1) |  | |  |
| **Baseline Ulcer Grade = 2** |  |  | |  |
| ITT, % (n) | 40.0% (20) | 18.2% (4) | | 21.82% |
|  |  |  | | (95% CI, 0.74%~42.89%) |
| PP, % (n) | 47.5% (19) | 21.1% (4) | | 26.45% |
|  |  |  | | (95% CI, 2.46%~50.44%) |
| **1.5 cm^2^ ≤ Ulcer Size < 25 cm^2^** |  |  | |  |
| ITT, % (n) | 38.1% (16) | 11.1% (2) | | 26.98% |
|  |  |  | | (95% CI, 6.33%~47.64%) |
| PP, % (n) | 45.5% (15) | 12.5% (2) | | 32.95% |
|  |  |  | | (95% CI, 9.48%~56.43%) |
| **Baseline Ulcer Grade ≤ 2** |  |  | |  |
| ITT, % (n) | 40.4% (21) | 18.2% (4) | | 22.20% |
|  |  |  | | (95% CI, 1.28%~43.12%) |
| PP, % (n) | 47.6% (20) | 21.1% (4) | | 26.57% |
|  |  |  | | (95% CI, 2.81%~50.32%) |
| **1.5 cm^2^ ≤ Ulcer Size < 25 cm^2^** |  |  | |  |
| ITT, % (n) | 38.6% (17) | 11.1% (2) | | 27.53% |
|  |  |  | | (95% CI, 7.09%~47.96%) |
| PP, % (n) | 45.7% (16) | 12.5% (2) | | 33.21% |
|  |  |  | | (95% CI, 10.08%~56.34%) |
| **Baseline Ulcer Grade = 3** |  |  | |  |
| ITT, % (n) | 23.8% (5) | 38.5% (5) | | -14.65% |
|  |  |  | | (95% CI, -46.76%~17.46%) |
| PP, % (n) | 31.3% (5) | 45.5% (5) | | -14.20% |
|  |  |  | | (95% CI, -51.38%~22.97%) |
| **1.5 cm^2^ ≤ Ulcer Size < 25 cm^2^** |  |  | |  |
| ITT, % (n) | 22.2% (4) | 36.4% (4) | | -14.14% |
|  |  |  | | (95% CI, -48.45%~20.17%) |
| PP, % (n) | 30.8% (4) | 44.4% (4) | | -13.68% |
|  |  |  | | (95% CI, -54.70%~27.35%) |

Supplemental Table 4. Time to complete ulcer closure according to treatment group in the PP population

|  | **ENERGI-F703** | **Vehicle** | ***P*-Value**  **LOGRANK** | ***P*-Value**  **WILCOXON** |
| --- | --- | --- | --- | --- |
| **All Subject** |  |  |  |  |
| PP, n  Q1 days (95% CI) | 72  67 (51.0 ~ 84.0) | 35  84 (70.0 ~ ) | 0.1077 | 0.0939 |
| **ABI ≤ 1.4** |  |  |  |  |
| PP, n | 69 | 34 | 0.1446 | 0.1172 |
| Q1 days (95% CI) | 67 (45.0 ~ 84.0) | 84 (70.0 ~ ) |  |  |
| **ABI > 1.4** |  |  |  |  |
| PP, n | 2 | 1 | 0.4795 | 0.4795 |
| Q1 days (95% CI) | 84 (84.0 ~ ) | - |  |  |
| **Foot Ulcers** |  |  |  |  |
| PP, n  Q1 days (95% CI) | 58  67 (45.0 ~ 84.0) | 30  84 (70.0 ~ ) | 0.1704 | 0.1102 |
| **Leg Ulcers** |  |  |  |  |
| PP, n  Q1 days (95% CI) | 14  69 (27.0 ~ 87.0) | 5  - (70.0 ~ ) | 0.3351 | 0.5256 |
| **Baseline Ulcer Grade ≤ 2** |  |  |  |  |
| PP, n  Q1 days (95% CI) | 50  58 (45.0 ~ 84.0) | 24  - (56.0 ~ ) | 0.0180 | 0.0158 |
| **1.5 cm^2^ ≤ Ulcer Size < 25 cm^2^** |  |  |  |  |
| PP, n  Q1 days (95% CI) | 41  67 (45.0 ~ 87.0) | 20  - (70.0 ~ ) | 0.0080 | 0.0078 |
| **Baseline Ulcer Grade = 3** |  |  |  |  |
| PP, n  Q1 days (95% CI) | 22  84 (28.0 ~ ) | 11  70 (39.0 ~ ) | 0.6885 | 0.6597 |
| **1.5 cm^2^ ≤ Ulcer Size < 25 cm^2^** |  |  |  |  |
| PP, n  Q1 days (95% CI) | 19  84 (42.0 ~ ) | 9  70 (39.0 ~ ) | 0.7419 | 0.6672 |

(1) ABI = ankle brachial index.

(2) ABI of a subject in ENERGI-F703 treatment group was unknown and excluded from the results.

(3) “-“ = not able to estimate Q1 days.

Supplemental Table 5. Time to complete ulcer closure excluding subjects with lower leg ulcer

|  | **ENERGI-F703** | **Vehicle** | **LOGRANK** | **WILCOXON** |
| --- | --- | --- | --- | --- |
| **All Subject** |  |  |  |  |
| ITT, n | 73 | 35 | 0.1770 | 0.1110 |
| Q1 days (95% CI) | 67 (51.0 ~ 84.0) | 84 (70.0 ~) |  |  |
| PP, n | 58 | 30 | 0.1704 | 0.1102 |
| Q1 days (95% CI) | 67 (45.0 ~ 84.0) | 84 (70.0 ~) |  |  |
| **Baseline Ulcer Grade ≤ 2** |  |  |  |  |
| ITT, n | 52 | 22 | 0.0361 | 0.0319 |
| Q1 days (95% CI) | 58 (45.0 ~ 84.0) | - (56.0 ~) |  |  |
| PP, n | 42 | 19 | 0.0367 | 0.0328 |
| Q1 (95% CI) | 58 (42.0 ~ 84.0) | - (56.0 ~) |  |  |
| **1.5 cm^2^ ≤ Ulcer Size < 25 cm^2^** |  |  |  |  |
| ITT, n | 44 | 18 | 0.0192 | 0.0182 |
| Q1 (95% CI) | 58 (45.0 ~ 84.0) | - (70.0 ~) |  |  |
| PP, n | 35 | 16 | 0.0197 | 0.0186 |
| Q1 (95% CI) | 58 (42.0 ~ 84.0) | - (70.0 ~) |  |  |
| **Baseline Ulcer Grade = 3** |  |  |  |  |
| ITT, n | 21 | 13 | 0.5455 | 0.6321 |
| Q1 (95% CI) | 84 (28.0 ~) | 70 (39.0 ~) |  |  |
| PP, n | 16 | 11 | 0.6001 | 0.6457 |
| Q1 (95% CI) | 77 (28.0 ~) | 70 (39.0 ~) |  |  |
| **1.5 cm^2^ ≤ Ulcer Size < 25 cm^2^** |  |  |  |  |
| ITT, n | 18 | 11 | 0.5637 | 0.5857 |
| Q1 (95% CI) | 84 (42.0 ~) | 70 (39.0 ~) |  |  |
| PP, n | 13 | 9 | 0.6092 | 0.6074 |
| Q1 (95% CI) | 84 (42.0 ~) | 70 (39.0 ~) |  |  |
